# The Role of Occupational Therapy in Pulmonary Rehabilitation Programs: Protocol for a Scoping Review

**DOI:** 10.2196/30244

**Published:** 2021-07-26

**Authors:** Natalie Snyder, Ria Wilson, Lian Finch, Brooklyn Gallant, Chris Landa, Daniel Frankel, Dina Brooks, Tara Packham, Ana Oliveira

**Affiliations:** 1 School of Rehabilitation Science Faculty of Health Sciences McMaster University Hamilton, ON Canada; 2 Respiratory Medicine West Park Healthcare Centre Toronto, ON Canada; 3 Rehabilitation Sciences Institute Temerty Faculty of Medicine University of Toronto Toronto, ON Canada; 4 Lab3R – Respiratory Research and Rehabilitation Laboratory School of Health Sciences University of Aveiro Aveiro Portugal; 5 Institute for Biomedicine University of Aveiro Aveiro Portugal

**Keywords:** chronic respiratory disease, COPD, lung diseases, occupational therapy, pulmonary rehabilitation program

## Abstract

**Background:**

Chronic respiratory diseases are highly prevalent and compromise an individual’s ability to perform activities of daily living (ADLs) and participate in meaningful life roles. Pulmonary rehabilitation (PR) is a well-established intervention aimed at restoring an individual’s exercise capacity and improving their ability to complete their ADLs. Occupational therapists help individuals engage in meaningful “occupations,” improving their health and well-being. Given the concordance in the aims of PR and the occupational therapy (OT) scope of practice, occupational therapists appear to be well suited as key players in PR programs. However, the benefits of adding OT to PR programs have been sparsely reported in the literature and the role of OT in PR has never been synthesized or reported in national and international guidelines.

**Objective:**

The aim of this review is to explore the role of OT in PR programs, the current guideline recommendations for the inclusion of OT in PR programs, the estimated prevalence of OT in PR programs, and the reported or anticipated effects of OT interventions in PR programs.

**Methods:**

The review will be conducted following the Joanna Briggs Institute (JBI) methodology for scoping reviews. A comprehensive search will be undertaken in the Cochrane Database of Systematic Reviews, EMBASE, MEDLINE, and CINAHL (EBSCO) to identify and retrieve relevant literature published in English, French, or Portuguese. Gray literature on international OT association websites will also be identified, including position statements and guidelines relevant to PR programs. All literature published since the establishment of the effectiveness of PR for chronic respiratory disease in 1994 that explores OT in PR programs for these patients will be included. Search results will be exported to Covidence for title, abstract, and full-text screening by two independent reviewers. Data will be extracted by two independent reviewers using a pilot-tested template including the following: the number of PR programs including OT (specifically from surveys), the purpose of the study, the study design, patient characteristics, respiratory conditions included, PR components, OT role, outcomes, and results. Findings will be presented using a narrative summary, supplemented by figures and/or tables. Key themes will be displayed in an infographic or schematic.

**Results:**

The study was initiated in January 2021 and registered with the Open Science Framework (OSF) in February 2021, prior to title and abstract screening. Data collection and analysis and drafting of the manuscript will occur throughout 2021, with expected publication in 2022.

**Conclusions:**

The results of this scoping review will help health care professionals improve patient care by broadening their understanding and awareness of the role of OT in PR programs. This role clarification may help to inform program development and clinical decision making and will serve to optimize the delivery of multidisciplinary care for patients in PR programs, ultimately improving patient outcomes.

**Trial Registration:**

OSF Registries ZH63W; https://osf.io/zh63w

**International Registered Report Identifier (IRRID):**

DERR1-10.2196/30244

## Introduction

Chronic respiratory diseases are chronic diseases of the airways and structures of the lung [1]. In 2017, an estimated 545 million people globally were impacted by chronic respiratory diseases; in Canada, approximately 1 in 10 adults are affected [2,3]. Chronic respiratory diseases impose a high burden of morbidity and mortality worldwide [2]. In recent years, chronic respiratory diseases represented the third-leading cause of death globally [2] and chronic obstructive pulmonary disease (COPD) represented the sixth greatest contributor to global disability-adjusted life years (DALYs) [4]. As such, people with chronic respiratory diseases present with multifaceted physical, psychological, and social impairments, compromising their ability to perform activities of daily living (ADLs) and threatening their participation in meaningful life roles [5].

Pulmonary rehabilitation (PR) is a core component of the management of respiratory diseases, and there is strong evidence to support its effectiveness in people with COPD [6], interstitial lung disease (ILD) [7], lung cancer [8], pulmonary arterial hypertension [9], and COVID-19 [10], as well as those who are pre–lung transplantation or post–lung transplantation [11,12], among others [6]. PR has been shown to relieve dyspnea and fatigue, improve overall exercise capacity, reduce hospital readmissions, enhance the sense of control that an individual has over their condition, and improve health-related quality of life [13,14]. A PR program generally involves a thorough individual assessment, followed by a comprehensive, multidisciplinary, person-centered intervention that may include education, exercise, and self-management strategies targeting healthy behavior change [6,15]. These interventions aim to restore an individual’s exercise capacity and improve their ability to complete ADLs [16].

Occupational therapists are rehabilitation professionals who work to help individuals engage in ADLs and other meaningful “occupations” that affect their health, well-being, and participation in life roles [16,17]. Given the concordance in the aims of PR and the occupational therapy (OT) scope of practice, occupational therapists appear to be well suited as key players in PR programs [18]. The benefits of adding OT to PR programs have been sparsely reported in small studies of patients with COPD [19-21] but have never been synthesized or reported in national and international guidelines [6,22]. The lack of knowledge about OT roles and benefits may be hampering its inclusion in PR programs and preventing people with chronic respiratory diseases from getting the best evidence-based care. Indeed, the most recent Canadian survey found that less than 40% of PR programs in Canada report having occupational therapists on their team [22].

In preparation for this review protocol, an initial search of the literature was conducted in EMBASE, MEDLINE, CINAHL, the Cochrane Database of Systematic Reviews, and the JBI Evidence Synthesis; no current or underway scoping reviews or systematic reviews on OT in PR were identified. However, the terms “occupational therapy,” “cardiopulmonary rehabilitation programs,” “respiratory rehabilitation programs,” and “pulmonary rehabilitation programs” yielded a wide breadth of literature. These preliminary search findings support the need for a scoping review to map the available research; explore the extent, range, and nature of the research activity; and identify gaps in the available evidence [23].

The main objective of this scoping review is to explore the role of OT in PR programs. The secondary objectives are the following: (1) systematically map the recommendations of current guidelines for the inclusion and roles of OT in PR programs, if any, (2) estimate the prevalence of OT as part of PR programs, and (3) summarize the reported effects of OT as part of PR.

## Methods

### Overview

The protocol is reported according to the Preferred Reporting Items for Systematic Review and Meta-Analysis Protocols (PRISMA-P) guideline [24]. The scoping review will be conducted in accordance with the JBI methodology for scoping reviews and will follow the Preferred Reporting Items for Systematic Reviews and Meta-Analyses extension for scoping reviews (PRISMA-ScR) for the reporting of results [25,26]. The protocol was registered with the Open Science Framework (OSF) from the Center for Open Science prior to title and abstract screening to improve research transparency and reduce risk of bias [27,28].

### Review Questions

This review will seek to answer the following questions:

1. What are the recommendations of current guidelines for the inclusion and roles of OT in PR programs, if any?
2. What is the estimated prevalence of OT in PR programs?
3. What are the reported effects of OT as part of PR?

### Inclusion Criteria

#### Participants

This review will consider literature pertaining to patients with chronic respiratory diseases who are receiving OT services in PR programs. Only studies in adult populations (aged ≥18 years) will be included to provide a more coherent summary of the available literature, since OT interventions differ significantly between children and adults. Additionally, pulmonary rehabilitation is currently primarily provided to adult patients and thus we estimate that literature in children will be too scarce for fulsome review. Chronic respiratory diseases include, but are not limited to, COPD, ILD, cystic fibrosis, bronchiectasis, and lung cancer.

#### Concept

This review will consider studies that explore the concept of OT in PR programs. These programs may be described as “cardiopulmonary rehabilitation programs,” “respiratory rehabilitation programs,” or “PR programs” in order to capture a wide range of possible interventions with similar therapeutic goals that may be described using different terms. To be considered for inclusion, studies have to report on PR programs as defined by Spruit and colleagues [6] (ie, delivered over a minimum of 4 weeks, and including exercise, education, and self-management or behavior change interventions) and must include and describe the role of OT in a PR program. An exception will be made for survey studies of PR programs, in which all surveys of PR programs will be included, even if OT is not part of the PR program or if programs surveyed did not meet the PR definition established. This decision was made since a preliminary search revealed that surveys on PR programs usually use a broader definition of PR, for example by including programs that only provide exercise training as well as programs established according to the international guidelines. Thus, including all surveys will allow more accurate conclusions about the prevalence of OT in PR programs. Additionally, if only surveys including OT were to be included it could skew the estimation toward an inflated estimation of OT inclusion in PR programs.

#### Context

To ensure the scope of the review has both breadth and depth, this review will consider studies where PR programs are delivered in institutional or noninstitutional settings including the home and community. Some context examples may be inpatient, outpatient, or home PR programs. Included studies will not be limited by sample location, culture, or race.

### Types of Sources

Quantitative, qualitative, and mixed methods study designs will be considered for inclusion, as well as clinical practice guidelines, systematic reviews, and PR program surveys. Where systematic reviews meet inclusion criteria at title and abstract screening, the individual studies included in the review will be screened for the eligibility criteria. The systematic review will only be included in data extraction if novel findings for the role of OT in PR programs are reported. Finally, gray literature will be searched from OT association websites for position statements and other practice guidance relating to PR. The OT association websites of Canada, the United States, Australia, and the United Kingdom were searched because these countries are pioneers and leaders in PR and thus more likely to have published guidelines on PR implementation. In addition, the OT association websites of Portugal, Brazil, and France were searched for consistency with our language inclusion criteria.

### Exclusion Criteria

The exclusion criteria were established a priori. Conference abstracts will not be included. Given the language competencies of the research team members, included literature may be published in English, Portuguese, and French. Studies published in any other languages will be excluded. Studies published prior to 1994 will be excluded, as prior to this time the efficacy of PR for the management of chronic lung disease was not yet established [29].

### Search Strategy

The search strategy will be collaboratively developed by the research team, in consultation with an experienced librarian at the McMaster University Health Sciences Library (Ontario, Canada). An initial limited search of the JBI Evidence-Based Practice Database, EMBASE, MEDLINE, and CINAHL (EBSCO) will be undertaken to identify articles on the topic. The text words contained in the titles and abstracts of relevant articles as well as the keywords and index terms used to describe the articles will be used to assist in developing the full search strategy. The search strategy, including all identified keywords and index terms, will be adapted for each database. Furthermore, the reference lists of articles selected for full-text review will be hand-searched for additional studies.

### Information Sources

Four databases will be searched: MEDLINE via Ovid (1946 to 2021), EMBASE via Ovid (1974 to 2021), CINAHL via EBSCOHost (1937-2020), and the Cochrane database (1995-2021). Multimedia Appendix 1 contains a complete description of search strategies for each database.

### Study Selection

Following the search, all identified records will be collated, uploaded into Zotero reference management software (Center for History and New Media, George Mason University), and duplicates removed. All records will then be uploaded to the Covidence screening and data extraction tool (Veritas Health Innovation). Two independent reviewers will perform a pilot test of the proposed eligibility criteria with 10 articles to achieve full understanding of the criteria and solve any disagreements before screening. Titles and abstracts will then be screened by two independent reviewers for assessment against the eligibility criteria for the review. Literature meeting the criteria will be retrieved in full and assessed against the eligibility criteria by two independent reviewers. Any disagreements that arise between the reviewers during title and abstract screening or full-text screening will be resolved through discussion with a third reviewer. Search results and reasons for exclusion of full-text papers that do not meet the eligibility criteria will be recorded and reported in a PRISMA-ScR flow diagram [26].

### Data Extraction

Data from papers included in the scoping review will be extracted to predefined tables by two independent reviewers. Tables will be created using the data extraction instrument from Peters and colleagues [30] adapted to address the review objectives and to take into consideration the different study designs [30]. Multimedia Appendix 2 contains full details of which data will be sought. The data extracted will include specific details describing the population, concept, and context of the study, as well as its methods and results relevant to our scoping review. A draft of the data extraction tables is provided in Multimedia Appendix 2. The draft data extraction tables will be modified and revised as necessary during the process of data extraction. Modifications will be detailed in the full scoping review. The first three references will be extracted together by two reviewers as a pilot test to ensure consistency and reliability. Data from all remaining studies will then be extracted by two independent reviewers and compared once all are complete, with a third reviewer available to consult in case of disagreement.

### Data Analysis and Presentation

The findings of the scoping review will be classified under conceptual domains based on the primary and secondary research questions and the Canadian Model of Occupational Performance and Engagement (CMOP-E) [31]. The CMOP-E was originally developed by the Canadian Association of Occupational Therapists and outlines the primary domains of interest to the scope of practice of OT. It has three components: (1) the person, situated at the center of the model, (2) the environment (the physical, cultural, institutional, and social spaces where the “occupations” occur), and (3) occupation, including the subcategories of self-care, productivity, and leisure. In this model, the occupation is seen as the connection between the person and their environment [31]. Describing the results in this manner will ensure clarity when reporting key findings. Furthermore, given that this model is consistent with the International Classification of Functioning Disability and Health (ICF), our findings will be presented in a manner that is in line with internationals standards [31]. A summary of extracted data will be provided in predefined tables (Multimedia Appendix 2). The extracted data will be reported in a narrative summary providing the answers to our research questions, and key findings will be presented in a table within the manuscript. We will also illustrate themes and their relationships in an infographic or schematic.

## Results

The project was initiated in January 2021. After formalizing the study methods, and prior to title and abstract screening, the protocol was registered with the Open Science Framework (OSF) from the Center for Open Science in February 2021. Data collection commenced in March 2021 and is ongoing at the time of submission. Data collection is expected to be completed by June 2021. Data analysis and drafting of the manuscript will occur throughout 2021, with expected publication in 2022.

## Discussion

To the author’s knowledge, this is the first scoping review to explore the role of OT in PR programs. By exploring a breadth of literature, including quantitative, qualitative, and mixed methods study designs, as well as clinical practice guidelines, systematic reviews, and PR program surveys, this study will provide a comprehensive description of the role of OT in PR programs. In addition, this study will systematically map the recommendations of current guidelines for the inclusion and roles of OT in PR programs, if any; estimate the prevalence of OT as part of PR programs; and summarize the reported effects of OT as part of PR. In doing so, this study will complement several guidelines that emphasize the importance of multidisciplinary care in PR [32-34] and may provide more specification and clarification for the role of OT in these multidisciplinary programs.

The results of this study will be of interest to all health care professionals and policy makers involved in the delivery of PR programs across many health care settings. The findings of this scoping review will allow these stakeholders to better understand the scope, roles, and responsibilities of OT within PR care pathways. This role clarification will inform program development and clinical decision making and will serve to optimize the delivery of multidisciplinary care for patients in PR programs, ultimately improving patient and health care services outcomes.

This proposed scoping review has many strengths. Preregistering the protocol prior to title and abstract screening reduces risk of bias and ensures research transparency [35]. Furthermore, using the JBI methodological framework and PRISMA guidelines for reporting results optimizes the rigor of this scoping review. Additionally, a comprehensive search strategy was developed in consultation with an experienced librarian, and the broad scope of the inclusion criteria was chosen to provide the most breadth and depth of information relating to the role of OT in PR programs. Finally, targeted gray literature searching will capture the most relevant information from OT association websites.

This scoping review is limited by language criteria, which restricts our ability to examine the role of OT in PR programs delivered in languages other than English, French, or Portuguese. There is a possibility that important roles of OT in PR programs exist in literature inaccessible to our group due to language barriers. Furthermore, though intentionally targeted, the search of gray literature will be minimal due to time constraints. Additionally, the literature search will be limited to four databases. It is likely that the majority of the relevant literature on the topic is included in these databases, but the possibility remains that additional literature of interest may be missed. Finally, and inherent to all scoping review methodology, this review will not include a critical appraisal of the evidence, which limits the ability of this review to make recommendations based on the literature or identify gaps on the basis of low-quality evidence.

In conclusion, this study will enhance our understanding of the role, extent, and effect of OT in PR programs, which has the potential for OT role enrichment or expansion, ameliorated interdisciplinary patient care and, most importantly, improved patient outcomes.

## Appendix

Multimedia Appendix 1 Search strategy.

Multimedia Appendix 2 Data extraction tables.

## Abbreviations

ADL: activities of daily living
CMOP-E: Canadian Model of Occupational Performance and Engagement
COPD: chronic obstructive pulmonary disease
DALY: disability-adjusted life year
ILD: interstitial lung disease
JBI: Joanna Briggs Institute
OSF: Open Science Framework
OT: occupational therapy
PR: pulmonary rehabilitation
PRISMA: Preferred Reporting Items for Systematic Reviews and Meta-Analyses
