# Supplementary material for: The Role of Occupational Therapy in Pulmonary Rehabilitation Programs: Protocol for a Scoping Review
Source: JMIR Res Protoc. 2021 Jul 26;10(7):e30244. doi: 10.2196/30244 (PMC8367120; doi:10.2196/30244)
Supplement: Multimedia Appendix 1 [file resprot_v10i7e30244_app1.docx]

## **Multimedia Appendix I: Search strategy**

EMBASE via Ovid search conducted on February 15, 2021

| Search | Query | Records retrieved |
| --- | --- | --- |
| #1 | lung disease.mp. OR exp lung disease/ OR chronic lung disease.mp. OR exp chronic lung disease/ OR COPD.mp. OR exp chronic obstructive lung disease/ OR interstitial lung disease.mp. OR exp interstitial lung disease/ OR exp asthma/ OR asthma. mp. OR cystic fibrosis.mp. OR exp cystic fibrosis/ OR bronchiectasis.mp. OR exp bronchiectasis/ OR lung cancer.mp. OR exp lung cancer/ | 1797574 |
| #2 | Exp occupational therapy/ OR occupational therap*.mp. OR (cardiopulmonary OR pulmonary OR respiratory) adj3 rehab* adj3 program*.mp.[mp=title, abstract, heading word, drug trade name, original title, device manufacturer, drug manufacturer, device trade name, keyword, floating subheading word, candidate term word] | 34984 |
| #3 | Pulmonary rehab* survey.mp. | 6 |
| #4 | 1 AND 2 | 2877 |
| #5 | 3 OR 4 | 2882 |
|  | Limited to English, French, and Portuguese | 2736 |

OT = Occupational Therapy, COPD = Chronic Obstructive Pulmonary Disease

Cochrane (CENTRAL) Database search conducted on February 17, 2021

| Search | Query | Records retrieved |
| --- | --- | --- |
| #1 | “Pulmonary Disease, Chronic Obstructive” [MeSH] OR “Asthma” [MeSH] OR “Lung Neoplasms” [MeSH] OR “Cystic Fibrosis” [MeSH] OR “Bronchiectasis” [MeSH] OR “Lung Diseases, Interstitial” [MeSH] OR “Lung Diseases” [MeSH] OR COPD[ti,ab,kw] OR “chronic obstructive pulmonary disease”[ti,ab,kw] OR asthma[ti,ab,kw] OR “lung cancer[ti,ab,kw] OR “cystic fibrosis”[ti,ab,kw] OR “interstitial lung disease”[ti,ab,kw] | 91628 |
| #2 | “Occupational Therapy” [MeSH] OR “Occupational Therapist” [MeSH] OR “occupational therapy”[ti,ab,kw] OR “occupational therapist”[ti,ab,kw] | 3409 |
| #3 | (cardiopulmonary or pulmonary or respiratory) NEAR/3 rehab* NEAR/3 program* | 844 |
| #4 | pulmonary rehab* survey | 0 |
| #5 | #2 OR #3 | 4247 |
| #6 | #1 AND #5 | 774 |

OT = Occupational Therapy, COPD = Chronic Obstructive Pulmonary Disease

CINAHL search conducted on February 16, 2021

| Search | Query | Records retrieved |
| --- | --- | --- |
| #1 | (MH “Lung Disease+”) OR “Lung Disease*” OR (MH “Lung Disease, Obstructive+”) OR “Chronic Lung Disease*” OR (MH “Pulmonary Disease, Chronic Obstructive+”) OR COPD OR Chronic Obstructive Pulmonary Disease” OR (MH “Lung diseases, interstitial+”) OR “Interstitial Lung Disease” OR (MH “Asthma+”) OR “Asthma” OR (MH “Cystic Fibrosis+”) OR “Cystic Fibrosis” OR (MH “Bronchiolitis+”) OR “Bronchiolitis” OR (MH “Lung Neoplasms+”) OR “Lung Cancer” OR “Lung Neoplasm” | 210,937 |
| #2 | (MH “Occupational Therapy+”) OR “Occupational Therap*” OR TX (cardiopulmonary or pulmonary or respiratory) n2 rehab* n2 program* | 50,394 |
| #3 | “Pulmonary Rehab* Survey” | 4 |
| #4 | #1 AND #2 | 690 |
| #5 | #3 OR #4 | 694 |
| Limited to: English, Portuguese, French | | 678 |

OT = Occupational Therapy, COPD = Chronic Obstructive Pulmonary Disease

Ovid Medline search conducted on February 16, 2021

| Search | Query | Records retrieved |
| --- | --- | --- |
| #1 | Lung Diseases/ OR chronic lung disease*.mp. OR exp Pulmonary Disease, Chronic Obstructive OR COPD.mp. OR exp Asthma/ OR asthma.mp. OR cystic fibrosis.mp. OR exp Cystic Fibrosis/ OR bronchiectasis.mp. OR Bronchiectasis/ OR lung cancer.mp. OR exp Lung Neoplasms/ OR exp Lung Diseases, Interstitial/ OR interstitial lung disease*.mp. | 1079241 |
| #2 | Exp Occupational Therapy/ OR exp Occupational Therapists/ or occupational ther*.mp. | 20691 |
| #3 | (pulmonary OR cardiopulmonary OR respiratory) adj3 rehab* adj3 program)mp. [mp=title, abstract, original title, name of substance word, subject heading word, floating sub-heading word, keyword heading word, organism supplementary concept word, protocol supplementary concept word, rare disease supplementary concept word, unique identifier, synonyms] | 1214 |
| #4 | Pulmonary rehab* survey.mp. | 4 |
| #5 | 2 OR 3 | 21892 |
| #6 | 1 AND 5 | 1309 |
| #7 | 4 OR 6 | 1311 |
| #* | Limit to French, Portuguese and English language | 1178 |

OT = Occupational Therapy, COPD = Chronic Obstructive Pulmonary Disease
