# Supplementary material for: The Role of Occupational Therapy in Pulmonary Rehabilitation Programs: Protocol for a Scoping Review
Source: JMIR Res Protoc. 2021 Jul 26;10(7):e30244. doi: 10.2196/30244 (PMC8367120; doi:10.2196/30244)
Supplement: Multimedia Appendix 2 [file resprot_v10i7e30244_app2.docx]

**Multimedia Appendix II: Data extraction tables**

| Experimental Studies | |
| --- | --- |
| Author, year, country |  |
| Study design |  |
| Groups |  |
| Sample size, gender, age |  |
| Respiratory Condition |  |
| Roles and outcomes of OT |  |
| Conclusion |  |

| Data Synthesis Studies | |
| --- | --- |
| Author, year, country |  |
| Purpose of the paper |  |
| Respiratory condition |  |
| Description of OT (goals) |  |
| Roles and outcomes of OT |  |
| Conclusions |  |

| Qualitative Studies | |
| --- | --- |
| Author, year, country |  |
| Approach |  |
| Participants, timing &  Respiratory Condition |  |
| Data Analysis methods |  |
| Sample size, sex, mean age (years) |  |
| Role of OT |  |
| Key findings for OT |  |

| Gray Literature | |
| --- | --- |
| Author, year, country |  |
| Respiratory Condition |  |
| Roles and Outcomes of OT in PR |  |

| PR Program Surveys | |
| --- | --- |
| Author, year, country |  |
| Purpose of the paper & response rate |  |
| Respiratory Condition included |  |
| Frequency, Roles and Outcomes of OT in PR programs |  |
| Results/ Conclusions about OT in PR |  |

 *Indicates the Canadian Model of Occupational Performance and Engagement (CMOP-E) will be used to code the OT intervention. OT = Occupational Therapy, PR = Pulmonary Rehabilitation
